# X-ray microtomography and linear discriminant analysis enable detection of embolism-related acoustic emissions

**DOI:** 10.1186/s13007-019-0543-4

**Published:** 2019-12-17

**Authors:** Niels J. F. De Baerdemaeker, Michiel Stock, Jan Van den Bulcke, Bernard De Baets, Luc Van Hoorebeke, Kathy Steppe

**Affiliations:** 1 Laboratory of Plant Ecology, Department of Plants and Crops, Faculty of Bioscience Engineering, Ghent University, Coupure links 653, 9000 Ghent, Belgium; 2 KERMIT, Department of Data Analysis and Mathematical Modelling, Faculty of Bioscience Engineering, Ghent University, Coupure links 653, 9000 Ghent, Belgium; 3 UGent-Woodlab-Laboratory of Wood Technology, Department of Environment, Faculty of Bioscience Engineering, Ghent University, Coupure links 653, 9000 Ghent, Belgium; 4 Ghent University Centre for X-Ray Tomography (UGCT), Proeftuinstraat 86, 9000 Ghent, Belgium; 5 Radiation Physics Group, Department of Physics and Astronomy, Faculty of Sciences, Ghent University, Proeftuinstraat 86, 9000 Ghent, Belgium

**Keywords:** Drought-induced embolism formation, Acoustic emissions, Machine learning, Linear discriminant analysis, X-ray computed microtomography, *Fraxinus excelsior* L.

## Abstract

**Background:**

Acoustic emission (AE) sensing is in use since the late 1960s in drought-induced embolism research as a non-invasive and continuous method. It is very well suited to assess a plant’s vulnerability to dehydration. Over the last couple of years, AE sensing has further improved due to progress in AE sensors, data acquisition methods and analysis systems. Despite these recent advances, it is still challenging to detect drought-induced embolism events in the AE sources registered by the sensors during dehydration, which sometimes questions the quantitative potential of AE sensing.

**Results:**

In quest of a method to separate embolism-related AE signals from other dehydration-related signals, a 2-year-old potted *Fraxinus excelsior* L. tree was subjected to a drought experiment. Embolism formation was acoustically measured with two broadband point-contact AE sensors while simultaneously being visualized by X-ray computed microtomography (µCT). A machine learning method was used to link visually detected embolism formation by µCT with corresponding AE signals. Specifically, applying linear discriminant analysis (LDA) on the six AE waveform parameters amplitude, counts, duration, signal strength, absolute energy and partial power in the range 100–200 kHz resulted in an embolism-related acoustic vulnerability curve (VC_AE-E_) better resembling the standard µCT VC (VC_CT_), both in time and in absolute number of embolized vessels. Interestingly, the unfiltered acoustic vulnerability curve (VC_AE_) also closely resembled VC_CT_, indicating that VCs constructed from all registered AE signals did not compromise the quantitative interpretation of the species’ vulnerability to drought-induced embolism formation.

**Conclusion:**

Although machine learning could detect similar numbers of embolism-related AE as µCT, there still is insufficient model-based evidence to conclusively attribute these signals to embolism events. Future research should therefore focus on similar experiments with more in-depth analysis of acoustic waveforms, as well as explore the possibility of Fast Fourier transformation (FFT) to remove non-embolism-related AE signals.

## Background

The strategy adopted by vascular plants to absorb and transport water through their conducting xylem tissue during transpiration could be described as brilliant, but lazy. Most other higher species use energy to transport sufficient amounts of water to sustain their metabolism. In contrast, plants exploit the gradient in water potential, from less negative to more negative, to enable water flow through their vascular tissue. Consequently, xylem vessels and tracheids are well adapted to withstand negative water potentials [1, 2], but in drying soil and/or atmospheric conditions, this passive strategy involves the risk of embolism formation, impairing the xylem conducting system [3]. Embolism causes water to be pulled from the vessel or tracheid and being replaced with air, resulting in the formation of emboli [4].

Embolism formation is accompanied by a sudden and rapid release in tension, producing energy waves detectable as acoustic emissions (AE) [5–7]. Milburn and Johnson [8] were the first to register acoustic emissions from a dehydrating leaf petiole, and linked these signals to embolism formation. The first commercially available AE counter to study AEs in wood was developed by Tyree and Sperry [9], and both AE sensors and acquisition systems have been greatly enhanced since then, allowing to record and analyze time, parameter and waveform data of each AE event [10–12]. Frequently used AE waveform parameters to study wood properties are peak amplitude, duration and energy [13], and when adding signal strength and partial power in the range 100–200 kHz [10, 12, 14], they have also been classified as important parameters related to embolism formation.

To quantify drought-induced embolism formation, a xylem vulnerability curve (VC) is typically constructed, relating loss of xylem water transport capacity to xylem water potential [15]. The standard hydraulic method to construct VCs is destructive and discontinuous, creating single VCs from extensive sampling [16]. In contrast, the continuous and non-invasive nature of the AE method allows (i) to develop sample-specific VCs [17], (ii) to characterize anatomical differences in thin drying wood sections [18], and (iii) to be used in outdoor applications [19]. This method can therefore be recommended as a valuable diagnostic tool to assess drought-induced embolism formation. However, aside from the findings reported by Tyree et al. [6] and Lewis [20], the assumption that all recorded AEs represent single embolism events [21] is in most cases invalid [11, 22]. Detecting embolism-related AE is therefore deemed necessary but remains challenging as a wide variety of AE sources is registered during dehydration, including water loss from other xylem elements such as fibers, tracheids and parenchyma [23, 24], mechanical strains [25, 26], dehydration of bark tissue [18], nanobubble formation [27], Haines jumps [12], and macro- and micro-crack formation [12, 28]. Because AEs do not quantify loss of hydraulic conductivity in the same way as the hydraulic method [15], the AE method has also been cited to be more qualitative than quantitative [16], making detection of embolism-related AE from total measured AE even more important. In order to unravel the link between acoustic emissions and drought-induced embolism formation, additional techniques are required that (i) can visualize the embolization process in order to delimit registered AEs within embolization time intervals, and that (ii) can deduce the acoustic characteristics of the signals in order to link AE to embolism formation.

In recent years, X-ray computed microtomography (µCT) has evolved from a niche technology [29] into an accessible reference visualization technique in drought-induced embolism research [30–32]. Reconstructed µCT images visualize the spatial distribution of the linear attenuation coefficient µ of the X-rays within the sample, implying that water-filled vessels appear as grey (high µ) and air-filled vessels as black (low µ) on the image [33]. The µCT method is therefore capable to non-invasively and continuously visualize embolism formation. It has significantly increased our understanding of drought-induced embolism formation, including (i) resolving issues related to artificial VCs of long-vesselled species constructed with indirect methods [30], (ii) providing evidence on the controversy of embolism refilling under tension [34, 35], and (iii) when vessel dimensions are extracted from which theoretical hydraulic conductance can be calculated, translating qualitative percentage cavitation into quantitative percentage loss of conductance (PLC) [32].

Machine learning has been proven to be an important diagnostic tool for processing, analyzing and deducing biological data [36, 37]. Machine learning is typically divided into unsupervised and supervised learning, where the former is used to model the data structure to identify hidden patterns in unlabeled datasets based on input variables [38], while the latter requires output variables to find a mapping function so that from new input data, output variables can be effectively predicted [38]. Unsupervised learning often starts with an exploratory procedure, like principal component analysis (PCA), to explain the data variance. Correlation matrix plots (i.e., correlograms) are complimentary tools to determine which variables are strongly positively or negatively correlated to one another. Histograms and receiver operating characteristic (ROC) curves can be used on PCA and correlogram results to visualize the underlying data distribution [39]. Supervised learning methods, such as linear discriminant analysis (LDA), incorporate output variables as labels during data analysis, which is more beneficial when specific patterns need to be extracted. LDA is a generative model [40], with the benefit that the model can be fitted efficiently, because the obtained parameters are directly computed from simple statistics such as average, variance and covariance.

We therefore conducted an experiment consisting of a combination of repeated 30-min rotation µCT imaging (6 × 4-min scans + 6-min break) with supervised LDA during continuous AE registration (with two AE sensors, one 14.0 cm (AE_1_) and one 24.3 cm (AE_2_) downstream from the scanning position to compare signal output) in a stem of a progressively dehydrating 2-year-old potted *Fraxinus excelsior* L. tree. Histograms of both embolism and non-embolism datasets, classified by µCT results, and ROC curves of the entire dataset were determined using the AE waveform parameters amplitude, counts, duration, signal strength, absolute energy and partial power in the range of 100–200 kHz to demarcate possible thresholds for these parameters, because these were, according to PCA, most suited for detecting embolism-related AE. The hypothesis is that if only embolism-related AE signals, instead of all registered AE signals, are used to construct acoustic VCs, these will correspond better with the reference VC_CT_ and, hence, improve the characterization of drought-induced vulnerability to embolism formation of species when using acoustic sensors.

## Results

### Unfiltered VC_AE_

**Fig. 1:**
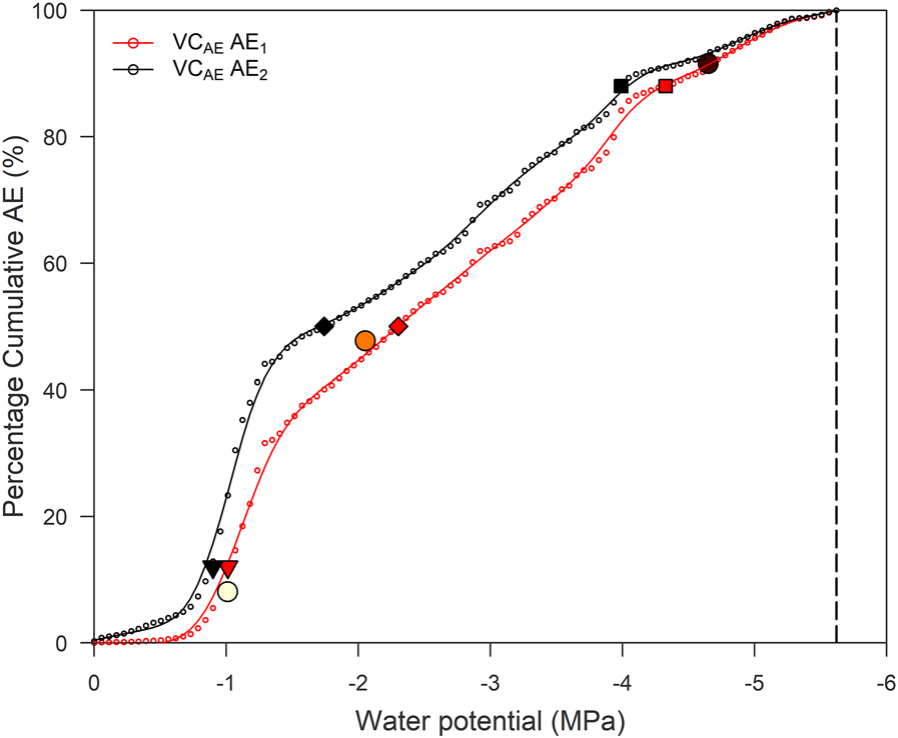
Acoustic vulnerability curve (VC_AE_) of *Fraxinus excelsior* L. during dehydration registered by sensor AE_1_ (red) and sensor AE_2_ (black). Vulnerability values AE_12_ (filled inverted triangle), AE_50_ (filled diamond), AE_88_ (filled square) and AE_100_ (dashed line) are indicated. LDA model outputs for embolism datasets 8, 69 and 120 (open circle) (Table 2), extracted from Fig. 4, are also shown

The VC_AE_s constructed from all AE signals measured by sensors AE_1_ and AE_2_ were similar in shape, with AE_2_ registering almost four times more signals (Fig. 1). Values obtained from VC_AE2_, especially AE_50_, were shifted to higher values of vulnerability to drought-induced embolism formation (Table 1).

**Table 1:** Vulnerability to drought-induced embolism values in *F. excelsior* L. derived from the acoustic vulnerability curve (VC_AE_) measured with sensor AE_1_ and sensor AE_2_

Vulnerability value (MPa)	AE_1_	AE_2_
AE_12_	− 1.02	− 0.88
AE_50_	− 2.30	− 1.73
AE_88_	− 4.32	− 3.98
AE_100_	− 5.62	− 5.62

Water potential at 12, 50, 88 and 100% cumulative acoustic emissions in *F. excelsior* L. (AE_12_, AE_50_, AE_88_, AE_100_) of sensor AE_1_ and sensor AE_2_

### Histograms and ROC curves

**Fig. 2:**
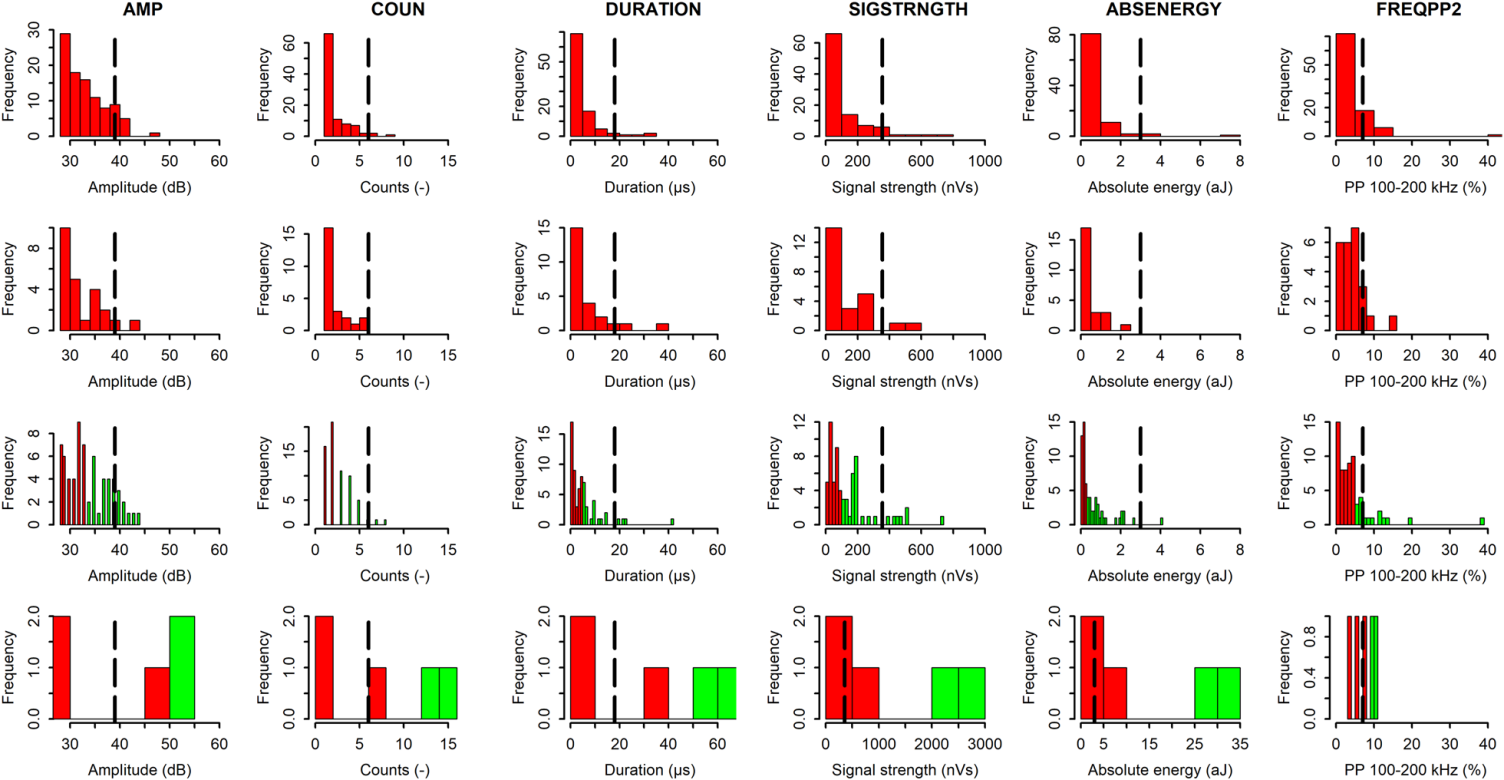
Histograms of the AE waveform parameters AMP (amplitude, dB), COUN (counts, −), DURATION (duration, µs), SIGSTRNGTH (signal strength, nVs), ABSENERGY (absolute energy, aJ) and FREQPP2 (partial power in the range 100–200 kHz, %) for non-embolism AE datasets (first two rows) and embolism AE datasets (last two rows). Retained AE signals in embolism datasets based on upper level histogram thresholding corresponding to the number of embolism registered by µCT are highlighted in green, and non-retained AE signals in both datasets in red. The black dashed line indicates the average threshold value for each parameter, with scaling adjusted in the case of outliers

To better link machine learning results of AE signals with µCT, analysis was conducted on the AE sensor closest to the µCT scanning point (AE_1_). Histogram plots of the AE waveform parameters peak amplitude (AMP), counts from peak amplitude (COUN), duration from peak amplitude (DURATION), signal strength (SIGSTRNGTH), absolute energy (ABSENERGY), and partial power in the frequency range 100–200 kHz (FREQPP2) (Table 4) in both embolism (green) and non-embolism (red) AE datasets showed that their upper level threshold values were most often associated with embolism events recorded by µCT (Fig. 2).

However, histogram results of AE_1_ signals illustrated that thresholds on AE parameters are insufficient to readily distinguish embolism signals (green) from non-embolism signals (red), as the thresholds sometimes included AE signals from non-embolism datasets and sometimes neglected AE signals from embolism datasets (Fig. 2). Thus, a static threshold on these six AE waveform parameters lacked sufficient accuracy.

**Fig. 3:**
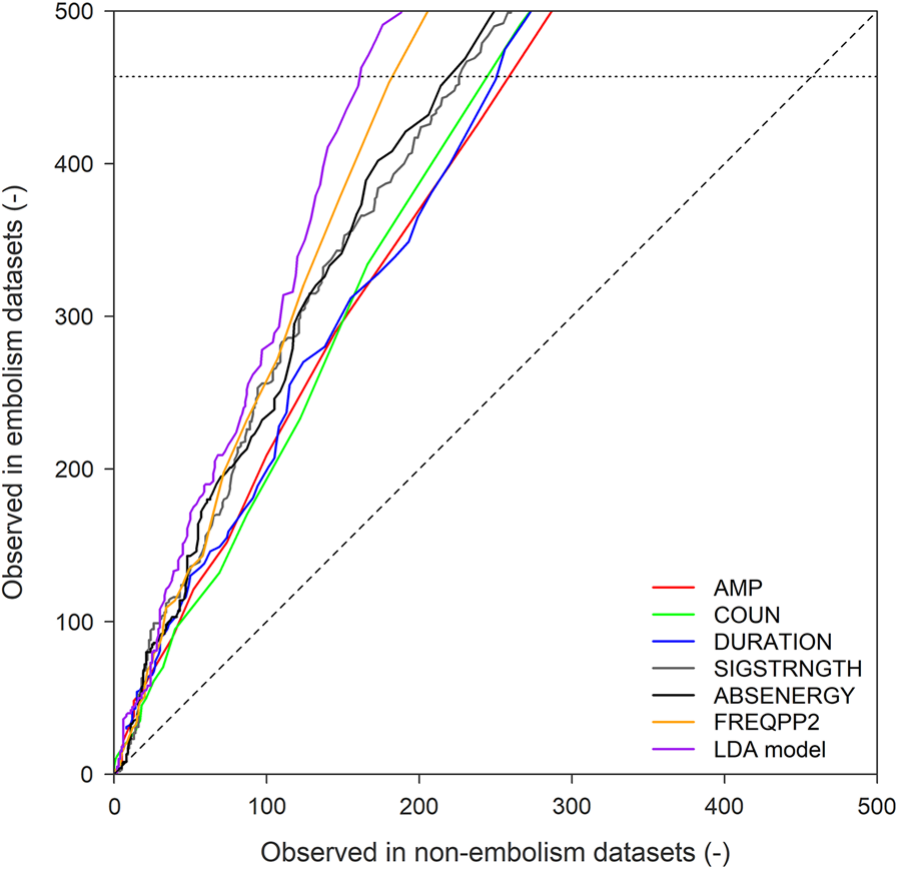
ROC curves of the AE waveform parameters AMP (red), COUN (green), DURATION (blue), SIGSTRNGTH (grey), ABSENERGY (black), FREQPP2 (orange) (Table 4) and linear discriminant analysis (LDA) model (purple) established for the AE dataset of sensor AE_1_. FREQPP2 and the LDA model produced the best ROC curves, because they were farthest away from the first bisector (dashed line) and closest to the number of embolism events registered by µCT (457, dotted line)

A static threshold based on the ROC curves of the AE waveform parameters AMP, COUN, DURATION, SIGSTRNGTH, ABSENERGY, and FREQPP2 (Table 4) for the entire AE_1_ dataset could not be determined, because all curves had a similar shape, showing no distinct deflection points (Fig. 3). Because the FREQPP2 ROC curve is farthest away from the first bisector, demarcating thresholds on this parameter would be most successful to detect embolism-related AE (Fig. 3), but the lack of a clear deflection point results in too much uncertainty, suggesting that a FREQPP2 static threshold at the 457 dotted line is insufficient to judge whether retained signals are related to embolism formation or not.

### LDA model

**Fig. 4:**
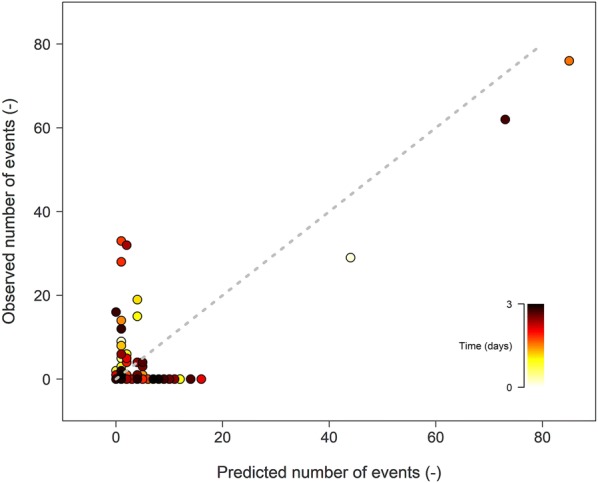
Linear discriminant analysis (LDA) modelling of the 132 AE datasets of sensor AE_1_ (time-gradient colored circles), relating LDA-predicted number of embolism events to µCT-observed number of embolism events

To determine which AE_1_ signals were related to embolism events, the LDA model was used with the six AE waveform parameters AMP, COUN, DURATION, SIGSTRNGTH, ABSENERGY, and FREQPP2 from the 132 AE_1_ datasets and the corresponding µCT embolism events as labels (Fig. 4). These results are obtained by using X-fold cross-validation. For each AE signal in the different datasets, LDA assigned a probability between 0 and 1. Per dataset, these probabilities were cumulated (predicted number of events) and compared to the number of embolism events detected by µCT (observed number of events).

Linear discriminant analysis yielded mixed results in detecting embolism-related AE: some AE datasets resulted in a close match between predicted and observed events, while other ones did not. The difference between predicted and observed events increased in function of dehydration time (Table 2).

**Table 2:** Number of embolism events according to X-ray computed microtomography (µCT) and linear discriminant analysis (LDA) model for the 132 AE datasets of sensor AE_1_

AE dataset	µCT	LDA	AE dataset	µCT	LDA
1–2	0–2	0–0	67–68	1–0	1–0
3–4	0–2	0–0	*69*–70	*76*–0	*85*–5
5–6	0–1	1–0	71–72	1–0	1–1
7–*8*	0–*29*	0–*44*	73–74	1–0	1–1
9–10	0–6	10–1	75–76	1–0	2–1
11–12	0–9	2–1	77–78	1–0	1–6
13–14	0–3	0–1	79–80	33–0	1–2
15–16	0–1	9–0	81–82	28–0	1–5
17–18	0–1	1–0	83–84	1–0	1–3
19–20	3–0	1–0	85–86	4–0	2–3
21–22	1–0	0–1	87–88	1–0	1–1
23–24	5–0	1–1	89–90	1–0	1–1
25–26	1–0	1–2	91–92	1–0	1–16
27–28	1–0	1–5	93–94	5–0	2–3
29–30	1–0	1–1	95–96	1–0	0–2
31–32	2–0	1–1	97–98	1–0	1–3
33–34	1–0	1–2	99–100	1–0	1–14
35–36	2–0	0–1	101–102	1–0	1–0
37–38	1–0	0–3	103–104	32–1	2–4
39–40	2–0	1–2	105–106	0–6	2–1
41–42	6–0	2–12	107–108	0–4	1–4
43–44	15–0	4–1	109–110	0–1	11–1
45–46	3–0	1–1	111–112	3–0	5–4
47–48	1–0	5–11	113–114	1–0	4–10
49–50	1–19	1–4	115–116	2–0	1–1
51–52	0–2	4–1	117–118	1–4	1–5
53–54	1–0	4–1	119–*120*	0–*62*	14–*73*
55–56	8–0	1–1	121–122	0–16	9–0
57–58	1–0	5–0	123–124	12–0	1–0
59–60	1–0	1–2	125–126	2–0	1–4
61–62	1–0	1–1	127–128	1–0	1–1
63–64	1–0	5–1	129–130	1–0	1–8
65–66	14–0	1–1	131–132	1–0	1–7

Italic values illustrate sufficient embolism-related AE detection by LDA in accordance with the number of embolism events by µCT. For every AE dataset, an LDA model was trained on all remaining datasets

Linear discriminant analysis shows promising results for larger AE datasets, which included a significant number of embolism events (datasets 8, 69 and 120; Table 2). These datasets were close to the first bisector (dashed line, Fig. 4), and corresponded well with the unfiltered VC_AE_ of sensor AE_1_ (Fig. 1). Furthermore, to detect embolism-related AEs, the ROC curve of LDA scored best compared to the ROC curve of the other six AE waveform parameters (Fig. 3).

Despite these positive indications, LDA probabilities attributed to AE signals were generally low (not higher than 0.4). This suggests that LDA might be a first promising step towards detecting embolism-related AE from an acquired AE dataset, yet not accurate enough.

### Embolism-related vulnerability

**Fig. 5:**
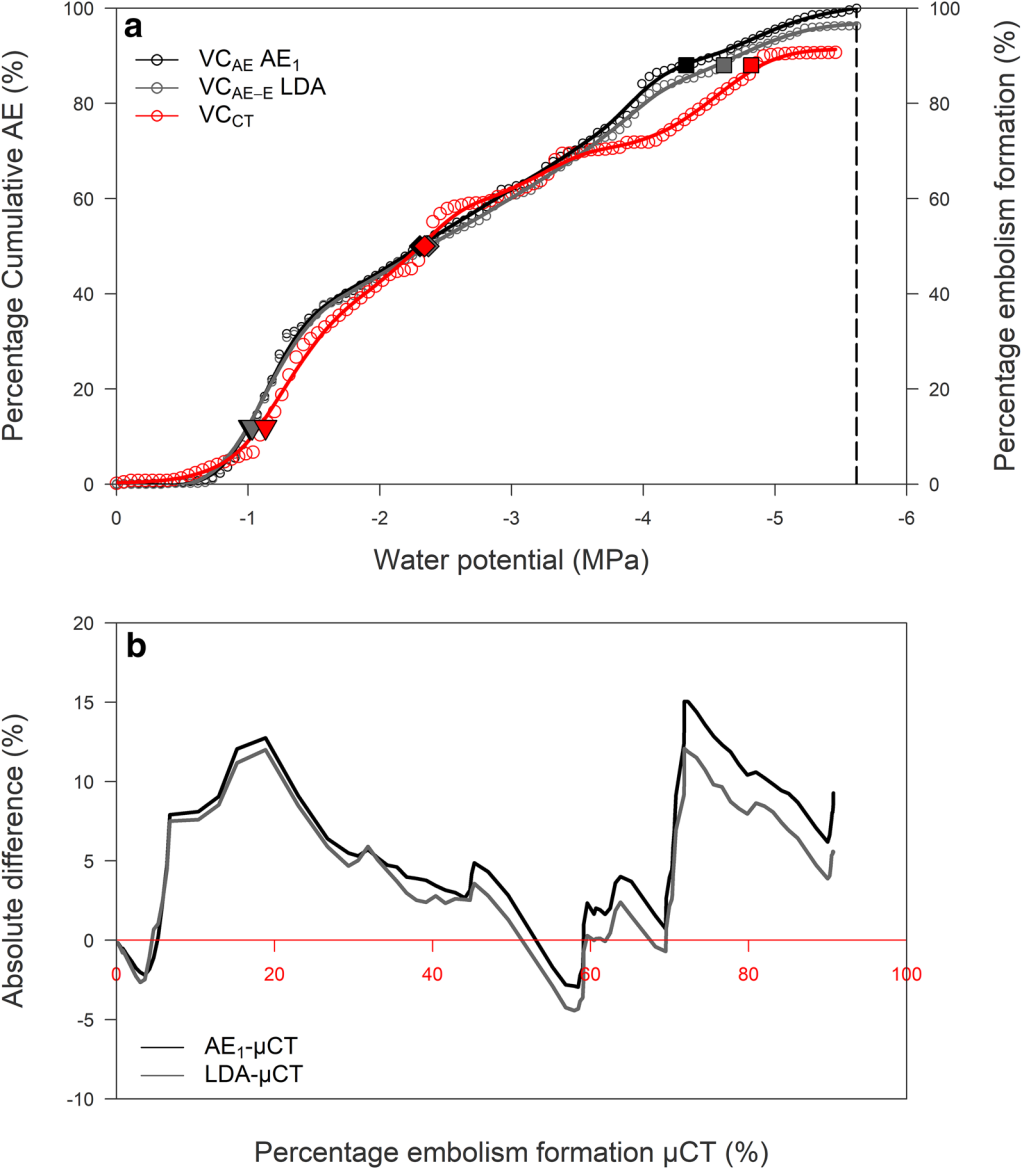
**a** Unfiltered acoustic vulnerability curve (VC_AE_ AE_1_, black), embolism-related VC using LDA (VC_AE-E_ LDA, grey) and µCT VC (VC_CT_, red) established for the dataset acquired by sensor AE_1_ on *Fraxinus excelsior* L. during dehydration. Vulnerability values AE_12_ (filled inverted triangle), AE_50_ (filled diamond), AE_88_ (filled square) and AE_100_ (dashed line) are indicated. **b** Absolute difference in percentage sembolism formation between AE_1_ (black) and µCT (red axis), and LDA (grey) and µCT (red axis). LDA filtering reduced the absolute difference in the effort to detect embolism-related AE

Comparing the embolism-related acoustic VC with the standard µCT VC (Fig. 5a) illustrates that LDA performs only a little better than the full acoustic VC in detecting embolism-related acoustic emissions mainly because of the over- and underestimation of LDA compared to the visually detected number of embolism events with µCT (Table 2). The absolute difference in percentage embolism formation to the reference VC_CT_ was calculated for VC_AE_ and VC_AE-E_, and was over the entire dehydration period slightly lower for the latter, mainly resulting in an overestimation of the number of embolism events compared to µCT (Fig. 5b).

Linear discriminant analysis detected a total of 518 embolism-related AE signals at the end of dehydration compared to 457 visually detected µCT embolism events (Table 2). Interestingly, the unfiltered VC_AE_ of sensor AE_1_, though registering a total of 25,901 AE signals at the end of dehydration, resulted in an AE_50_ that only slightly underestimated CT_50_ with 2% (Table 3; Fig. 5a, b). Divergence in vulnerability was mainly concentrated towards the end of the curve.

**Table 3:** Vulnerability-to-cavitation values for *F. excelsior* L. derived from the acoustic vulnerability curve (VC_AE_) of sensor AE_1_, the embolism-related VC (VC_AE-E_) based on LDA and the X-ray computed microtomography VC (VC_CT_)

Vulnerability value (MPa)	VC_AE_ AE_1_	VC_AE-E_	VC_CT_
AE_12_/CT_12_	− 1.02	− 1.03	− 1.13
AE_50_/CT_50_	− 2.30	− 2.37	− 2.34
AE_88_/CT_88_	− 4.32	− 4.61	− 4.82

Water potential at 12, 50 and 88% cumulative acoustic emissions (AE_12_, AE_50_ and AE_88_), and 12, 50 and 88% µCT detected embolism formation (CT_12_, CT_50_ and CT_88_) for *F. excelsior* L. of sensor AE_1_, LDA and µCT

## Discussion

### Detection of embolism-related AE to improve VC_AE_

Applying LDA on the six AE waveform parameters amplitude, counts, duration, signal strength, absolute energy and partial power in the range 100–200 kHz and µCT scanning to generate model labels was successful at detecting embolism-related AE events from the dataset both in number and over time (Table 2; Fig. 5). The resulting VC_AE-E_ closely corresponded to VC_CT_ (Table 3; Fig. 5), which is considered the reference to quantify a species’ vulnerability to drought-induced embolism formation [30]. Hydraulic P_50_ (i.e., xylem water potential at 50% loss of hydraulic conductivity) derived from the VC established by Lemoine et al. [41] for 1–3 year-old branches of well-watered 15–20 year-old *Fraxinus excelsior* trees was equal to − 3 MPa, which agreed well with the CT_50_-value of our 2-year-old stem (Table 3). The small difference between the branches and our stem might be attributed to the different techniques used (hydraulic vs µCT). Another reason could be linked to the hydraulic segmentation hypothesis that postulates that angiosperm trunks/stems (30–40 cm diameter) are 0.7–1.8 MPa more vulnerable than branches (8–14 cm diameter) [42], but is less likely because of the close resemblance in our stem diameter to the investigated branch diameter of Lemoine et al. [41].

The close agreement between LDA VC_AE-E_ and VC_CT_ does however not imply that embolism-related AE signals can be readily distinguished from other AE sources, because the probability of the LDA model attributed to each AE signal was never higher than 0.4. Cumulative probabilities of all AE signals in embolism and non-embolism datasets were indicative for the number of µCT embolism events (Table 2), but cumulative probabilities of only AE signals detected by LDA (i.e., signals with highest probabilities according to LDA) were not, resulting in only 16 embolism events instead of the detected 457. Uncertainty remains whether AE signals with high values of amplitude, counts, duration, signal strength, absolute energy and partial power in the range 100–200 kHz are indeed typical characteristics of drought-induced embolism events, because these signals also occur in non-embolism AE datasets (Figs. 2, 3, 5b).

Acoustic emissions waveform parameters amplitude, duration, energy, signal strength and partial power in the range 100–200 kHz have previously been associated with embolism formation [10, 12, 14]. We showed that detection based on static thresholds for these parameters did not work, and included non-embolism AE sources (Fig. 2) [11]. This failure in using static thresholds can be attributed to species-specific AE attenuation in wood [13], which is known to decrease with ongoing dehydration [43]. AE sources registered at the start of dehydration will be more attenuated (due to the availability of more water) than at the end, and this attenuation factor is not taken into account when static thresholds on AE waveform parameters are used. Setting the threshold too low might include non-embolism-related AE measured at the end of dehydration, and setting the threshold too high might neglect the embolism events at the start of dehydration (Figs. 2, 3). Determination of dynamic thresholds that vary with time and incorporate changing attenuation with dehydration might enable separation of embolism from other AE-related sources during dehydration.

The unfiltered VC_AE_, though constructed from 25,901 cumulated AE signals, was closely related to VC_CT_ (Fig. 5a), both in magnitude and derived vulnerability characteristics (Table 3; Fig. 5b). This offsets the often-misplaced perception of AE being more qualitative, because of the excess of AE signals over embolism events registered during dehydration [16]. Compared to VC_CT_, this unfiltered VC_AE_ resulted in an AE_50_ which underestimated CT_50_ with only 2%. Incorporation of all AE signals for *F. excelsior* in a VC and using the VC_AE_ endpoint determination of Vergeynst et al. [12] produced a quantitative instead of a qualitative VC. This suggests that all AE signals registered during dehydration can be used to reliably assess drought vulnerability when compared to filtered AE methods [10–12] or reference techniques (hydraulic and µCT) [6, 7, 14, 44, 45].

### Significance of sensor installation to detect embolism-related AE

To reduce the number of AE signals not originating from embolizing conducting elements, sample length (with respect to maximum vessel length) and position of the sensor must be well-conceived [46]. Because *F. excelsior* was dehydrated by exposing the root system, and not by cutting the stem (classic way), maximum vessel length was not an issue in our study, and also cutting artifacts were avoided [16]. However, sensor installation did affect the number of registered AE signals (i.e., 25,901 for AE_1_ vs. 90,416 for AE_2_) and shifted VC_AE2_ to a slightly higher vulnerability to drought-induced embolism formation (Table 1; Fig. 1). The difference in AE registration was attributed to the installation position of sensor AE_2_, which was just below a leafy non-lignified side branch (Fig. 7), and closer to the tree’s foliage than sensor AE_1_ (i.e., 22.5 cm for AE_1_ vs. 11.7 cm for AE_2_). With the frequency of acoustic waves changing on their path through the wood towards the sensor [11, 43], less attenuation of AE sources originating from dehydrating leaves and side branches occurred in AE_2_, resulting in a higher number of detected AE signals above the noise threshold of 28 dB. The higher noise to embolism ratio of sensor AE_2_ alongside the closer position of sensor AE_1_ to the µCT scanning point further explains why results of sensor AE_1_ were used for machine learning analysis.

The high attenuation factor of wood, especially at the start of dehydration, significantly influences amplitude, frequency, shape-related and energy-related characteristics of registered AE signals [13]. If the effect of distance to the AE sensor is not quantified, then any classification approach can incur a large error. It is recommended that the effect of attenuation and its evolution during dehydration is quantified to reduce these errors as much as possible. This requires a mapping of the localization area of AE signals originating from the sample, which can be achieved by installing multiple AE sensors at known distances alongside the sample [13].

Despite the 71% difference in acquired AE signals between sensors AE_1_ and AE_2_, the VC_AE_s were similar in shape (Table 1; Fig. 1). Nonetheless, AE_2_ was less suited to identify the embolism-related signals, because to successfully detect embolism-related AE via LDA, the embolism to non-embolism signal ratio must be maximized. The embolism to non-embolism signal ratio was already low in sensor AE_1_ (25,444 non-embolism signals vs. 457 embolism signals), and increased dramatically in AE_2_, explaining why resulting histograms, ROC curves and LDA of sensor AE_1_ (Figs. 3, 4) showed difficulties in distinguishing embolism from other AE sources. Sensors should therefore be installed at a sufficiently large distance from the leaves, and in case cut branches or stems are used, sufficiently far from the open cut end of the sample.

### Maximum in third derivative to define the VC_AE_ endpoint

**Fig. 6:**
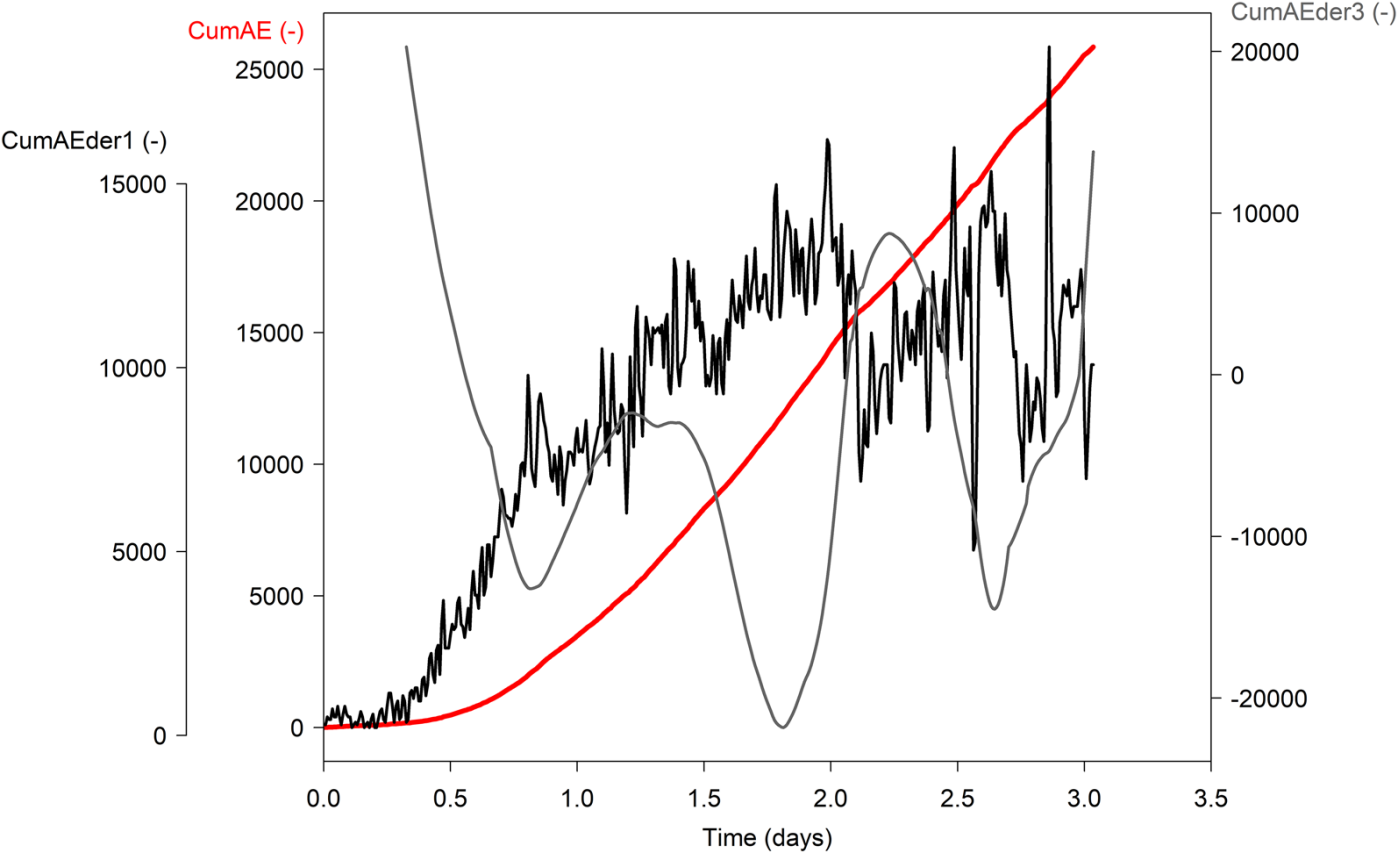
Average cumulative acoustic emissions (CumAE, red; 10-min averages), first derivative or AE activity (CumAEder 1, black; moving window interval: 15 min) and third derivative (CumAEder3, grey, moving window interval: 48 h) of *Fraxinus excelsior* L. registered by sensor AE_1_ during dehydration. The endpoint of VC_AE_ corresponds to the local maximum of the third derivate after the maximum in AE activity, which was not yet reached as indicated by the continuous increase in third derivative at the end of the acoustic curve

The µCT image taken at the end of the experiment (Fig. 11b) showed that 9% of the vessels were not embolized. The AE datasets therefore did not include the true VC_AE_ endpoint, and all registered signals were used to construct VC_AE_, and to detect embolism-related AE signals. In general, the VC_AE_ endpoint is defined by the local maximum of the third derivative of cumulative AE [12]. Because AEs are still recorded after full embolism formation due to a variety of other AE sources related to dehydration [12, 18, 23‒28], defining the endpoint of VC_AE_ remains a difficult and challenging task [47], but is crucial to derive physiologically-meaningful vulnerability characteristics. Vergeynst et al. [12] explained in their study that the time of reaching the maximum in the first derivative or AE activity can be used to define the time interval for calculating the third derivative (Fig. 6). As maximum AE_1_ activity occurred around 2 days, the third derivative was calculated with a time interval of 48 h. Because the resulting third derivative after the maximum in AE activity kept increasing, the local maximum was never reached for AE_1_. This agrees with the µCT results, which showed that 9% of the vessels were still functional when finishing the last CT-scan, and hence indirectly supports the VC_AE_ endpoint determination described by Vergeynst et al. [12]. Furthermore, xylem water potential registered at maximum AE activity (AE_50_ = − 2.58 MPa) coincided with CT_50_ (Table 3) [see 44], and further supports the soundness of Vergeynst et al.’s [12] endpoint determination for VC_AE_.

### Future perspectives for embolism-related AE detection

Detection of embolism-related signals from an AE dataset based on LDA modelling using the parameters amplitude, counts, duration, signal strength, absolute energy and partial power in the range 100–200 kHz was promising in the sense that the resulting VC_AE-E_ closely corresponded to the reference VC_CT_ in *F. excelsior* L. (Table 3; Fig. 5), and that the amount of AE signals to construct the VC were efficiently reduced (from 25,901 to 518 signals). However, the low embolism probabilities attributed by LDA to AE signals, as well as the time divergence in embolism formation detection (microseconds for AE vs 4 min for µCT), hamper the detection of distinct embolism-related AEs from acquired datasets. Multiple combined AE-µCT experiments on similar samples (age, species, treatment) can provide the necessary training datasets for LDA to better distinguish embolism from non-embolism AE sources, in order, for LDA, to be able to detect embolism-related AE, independent of µCT measurements, even for species for which no previous training datasets were acquired.

The non-invasive and continuous nature of AE sensors can also be applied to detect other physiologically meaningful AE sources from a dataset. When acoustically measuring and continuous scanning intact and well-watered trees, AE originating from shrinkage and water loss of fibers, tracheids and parenchyma can be captured [12, 23, 24]. Given the theory of water transport dynamics in plants, this shrinkage pattern occurs on a daily basis in well-watered plants, as a result of the time-lag that exists between foliar transpiration and root water uptake [48–50]. Using µCT scanning to verify that embolism formation does not occur, parameters of the registered AE signals can be analyzed and classified as non-embolism signals. This subset of AE signals could then be removed from acquired AE datasets, hence increasing the embolism to non-embolism signal ratio, and increasing the efficiency of supervised machine learning tools such as LDA to detect embolism-related AE.

## Conclusion

Ever since their first use in drought vulnerability research, acoustic emissions have been considered as interesting but unrefined to determine drought-induced embolism formation. The surplus of AE signals registered during dehydration not originating from embolism formation hinders correct quantitative assessment. Utilizing machine learning together with recorded embolism events by µCT scanning was proposed as a new method to detect embolism-related AE from an AE dataset gathered in a 2-year-old *F. excelsior* L. tree during progressive dehydration. LDA modelling based on the parameters amplitude, counts, duration, signal strength, absolute energy and partial power in the range 100–200 kHz was found sufficient to detect embolism-related AE probabilities that corresponded well with the µCT reference ones, but retained signals were still not easy distinguishable from other AE sources. Interestingly, the unfiltered acoustic VC resulted in vulnerability values that were in close agreement to the ones derived from the µCT VC, hence illustrating for this 2-year-old *F. excelsior* L. tree that unfiltered AE with the third derivate end point determination technique is accurate to determine its vulnerability to drought-induced embolism formation. Future research can still aim at a more in-depth analysis of acoustic waveforms and parameters associated with embolism formation to develop post-processing machine learning tools or state-of-the-art AE sensors that can efficiently filter embolism-related AE signals, without the aid of µCT. This will further promote the AE method as a reliable and quantitative, powerful diagnostic tool in future drought stress experiments.

## Methods

### Plant material and experimental setup

**Fig. 7:**
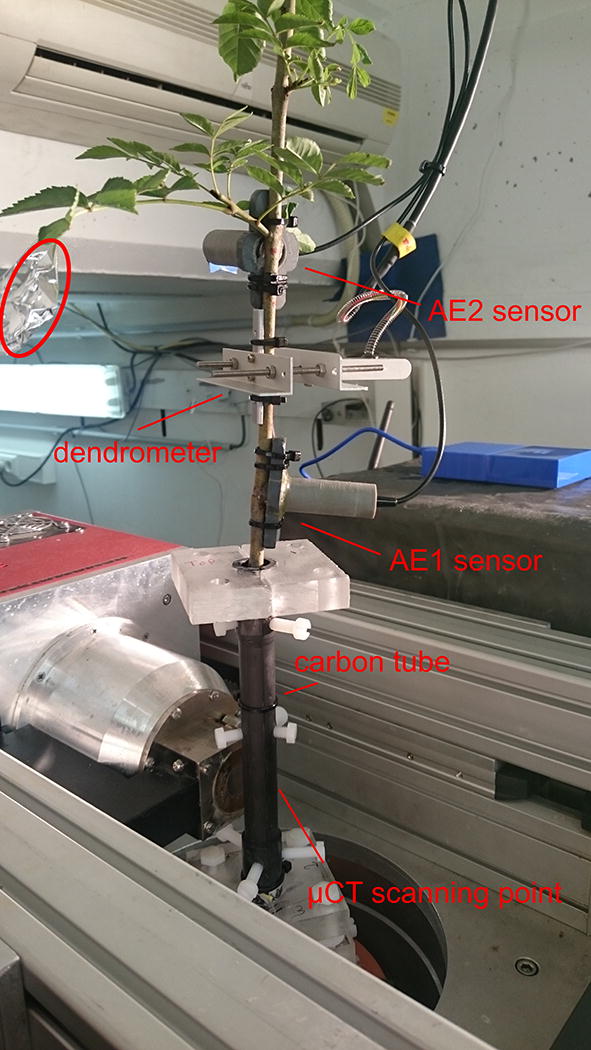
Experimental setup of the *Fraxinus excelsior* L. tree in the environmental µCT (EMCT) scanner. The EMCT continuously rotates around the stationary tree without the risk for twisting and winding of sensor cables. The carbon fiber tube was designed to ensure stable and centered positioning of the tree during µCT scanning. The tree is equipped with two broadband point-contact AE sensors in a pvc spring-containing holder to continuously register AEs from the progressively dehydrating xylem (AE_1_, AE_2_), and a point dendrometer to continuously register xylem shrinkage. The red circle indicates an aluminum enclosed leaf used for pressure chamber measurements to determine xylem water potential

Ten 2-year-old *Fraxinus excelsior* L. trees were grown in 3 L pots containing a soil mixture of peat litter, sand and calcium-magnesium based fertilizers in the greenhouse facilities of Ghent University (51° 03′ 10.3″ N latitude; 3° 42′ 32.3″ E longitude). Trees were grown under well-watered conditions for 2.5 months (from DOY 68 to DOY 142) during the 2017 growing season. On DOY 139, the tree with the straightest stem was selected for the measurement campaign, and replanted in a custom-built holder designed to keep the tree straight, centered, and tightly fixed during X-ray computed microtomography (µCT) scanning (Fig. 7; see Additional file 1). The tube enclosing the part of the tree that was scanned was made of carbon fiber (CarbonWinkel.nl, Tilburg, The Netherlands). On DOY 142, the tree was transported to the UGent Centre for X-ray Tomography (UGCT, https://wwsw.ugct.ugent.be), Belgium (51° 01′ 25.7″ N latitude; 3° 44′ 26.2″ E longitude), where the dehydration experiment took place from DOY 142 till DOY 145.

The tree was first removed from the custom-built holder to wash off the soil mixture, exposing the roots to speed up dehydration during scanning. The tree was re-inserted into the holder, and the loss in root anchoring countered by filling the excess room surrounding the roots with packaging foam. The tree was equipped with two broadband point-contact AE sensors with a flat frequency response between 20 and 1000 kHz (KRNBB-PC, KRN Services, Richland, WA, USA), at a respective distance of 14.0 cm (AE_1_) and 24.3 cm (AE_2_) downstream from the scanning position. The diameter of the tree, measured with an electronic caliper, was 6.6 mm at the AE1 sensor, and 6.3 mm at the AE_2_ sensor. At the position of the AE sensors, a section of bark (0.5 × 1.5 cm) was removed with a scalpel to expose the xylem, ensuring a better acoustic coupling with AEs originating from embolizing vessels [10]. To seal the wound and ensure good acoustic coupling, a droplet of vacuum grease (High-Vacuum Grease, Dow Corning, Seneffe, Belgium) was applied between sensor tip and xylem [45]. A compression spring (D22050, Tevema, Amsterdam, The Netherlands) in a small pvc tube was used to press the AE sensors against the xylem. To monitor xylem shrinkage, an additional section of bark (0.5 × 1.5 cm) was removed between the two AE sensors, the wound was sealed with petroleum jelly to prevent evaporation, the initial diameter (6.4 mm) was measured and the point dendrometer (DD-S, Ecomatik, Dachau, Germany) was installed at a distance of 19.2 cm downstream from the scanning position (Fig. 7; see Additional file 1).

The equipped tree was mounted on the z-stage of the Environmental µCT scanner (EMCT), a CT scanner custom-built by the Radiation Physics group (Fig. 7) [51]. This scanner is unique in its operating procedure, because X-ray tube and detector rotate around the stationary sample, opposite to most lab-based µCT scanners where it is the sample that rotates. As such, the EMCT allows objects to be equipped with peripheral sensors and equipment while still allowing for continuous CT scanning with a maximum rotation speed of one full rotation per 12 s. The scanner is controlled by a LabView interface [52]. See Dierick et al. [51] for more details about the set-up.

Distance between tree and X-ray source was 27 mm (Fig. 7), and the distance between X-ray source and detector 364 mm. The tube voltage was 70 kV, the tube power 8.47 W and no additional filtering was applied. A total of 7200 projections, with an exposure time of 200 ms per projection, were taken over six consecutive full rotations (1200 projections per rotation), with each rotation lasting 4 min, resulting in a total scan run duration of 24 min.

Between each run the scanner was paused for 6 min during the day and 30 min during the night to prevent overheating of the X-ray tube. A total of 15 runs was executed during daytime and 8 runs during evening and nighttime, with the exceptions of DOY 142 with 8 daytime runs (scanner and tree setup preparation), and DOY 145 with 6 daytime runs (end of experiment, including dismantling of the set-up). Reconstructions were automated using a Python wrapper for the Octopus reconstruction [53] software package (currently distributed by TESCAN-XRE, formerly known as XRE, spin-off company of UGCT), and resulted in a 3D reconstruction of a 7.5 mm section of the tree. The reconstructed data consisted of a total stack of 1000 reconstructed 2D slices, and an approximated voxel pitch of 7.5 µm was obtained.

During the daytime pauses of the EMCT scanner, measurements of xylem water potential (ψ_x_, MPa) were collected with the pressure chamber (Model 1000, PMS Instrument Company, Corvallis, OR, USA). Leaves excised for ψ_x_ measurements were wrapped in aluminum foil for at least 1 h to ensure equilibration between leaf and stem water potential (Fig. 7). During wrapping and excision, AE detection was put on hold to avoid noise disturbance.

Acoustic emissions sensors and dendrometer were connected to their respective data acquisition systems to enable continuous registration. Dendrometer read-outs were registered every minute via a custom-built acquisition board. The AE signals were amplified by 35.6 decibels (dB) with an amplifier (AMP-1BB-J, KRN Services, Richland, WA, USA) and waveforms of 7168 samples length were acquired at 10 MHz sample rate. The signals were collected using two 2-channel PCI boards and redirected to the software program AEwin (PCI-2, AEwin E4.70, Mistras Group BV, Schiedam, The Netherlands). A 20–1000 kHz electronic band pass filter was applied and only waveforms above the noise level of 28 dB were retained [12]. AE sensor installation was validated by the pencil lead break test [9, 43, 54]. Each collected AE signal was represented by a total of 18 AE waveform parameters (Table 4), with AE waveform parameters peak amplitude, rise time, counts from peak amplitude, wave energy, and duration from peak amplitude describing the shape of the AE signal (see Additional file 2). Internal clocks of dendrometer and AE acquisition systems were also matched with the EMCT to avoid differences in time between the datasets.

**Table 4:** AE waveform parameters of AE signals collected with the software program AEwin, including their respective abbreviation and unit

AE parameter	Abbreviation	Unit
Rise time	RISE	µs
Counts from peak amplitude	COUN	–
Wave energy	ENER	10–14 V2 s
Duration from peak amplitude	DURATION	µs
Peak amplitude	AMP	dB
Absolute frequency	AFRQ	kHz
Root mean square voltage	RMS	µV
Average signal level	ASL	dB
Reverberation frequency	RFRQ	kHz
Initiation frequency	IFRQ	kHz
Signal strength	SIGSTRNGTH	10–9 V s
Absolute energy	ABSENERGY	aJ
Partial power 0–100 kHz	FREQPP1	%
Partial power 100–200 kHz	FREQPP2	%
Partial power 200–400 kHz	FREQPP3	%
Partial power 400–800 kHz	FREQPP4	%
Frequency centroid	FRQC	kHz
Peak frequency	PFRQ	kHz

### Processing and linking µCT images to AE signals

**Fig. 8:**
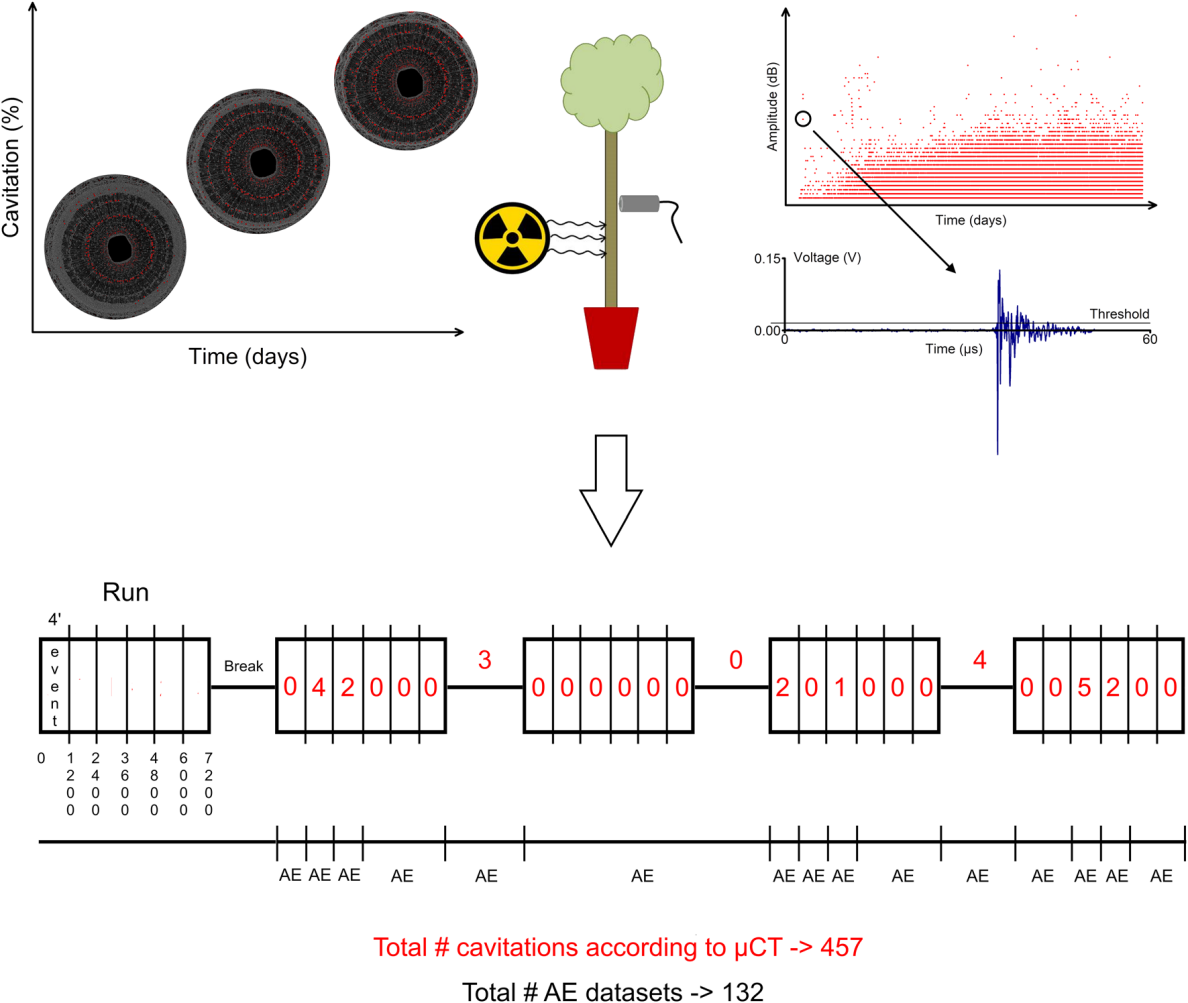
Schematic representation of how µCT images were processed and linked to AE signals. The left graph illustrates how embolism (red, %) spreads throughout 2D µCT cross-sections of *Fraxinus excelsior* L. as function of time (days). One stack consisting of 1000 2D µCT cross-sections resulted from reconstruction of 1200 projections collected over a time period of 4 min (event). Scans were consecutively taken over 24 min, resulting in one total scan run consisting of six events (i.e., 0–1200; 1200–2400; 2400–3600; 3600–4800; 4800–6000; 6000–7200; note that the number of projections per event are vertically displayed). A break was included between each run, and lasted 6 min during daytime runs and 30 min during evening and nighttime runs. The right graph shows the amplitude (dB) of all AE signals registered by sensor AE_1_ (closest to the scanning position) during progressive dehydration of the *Fraxinus excelsior* L. tree (time, days). Each dot in this graph represents the amplitude of one AE signal collected during dehydration. For each event and for the breaks between runs, µCT images were compared and analyzed for their total number of visually detected embolisms (red numbers), which totaled 457 at the end of the experiment. AE signals were grouped according to the time spans where embolism was detected or not detected, which resulted in 132 embolism and non-embolism AE datasets

For each event and for the breaks between runs, 50 mid-centered 2D slices were extracted from the total stack (1000 2D slices), combined and reconstructed into single µCT images, which were pairwisely compared and the number of visually detected embolisms quantified by the Fiji macro (Fig. 8). Each time an embolism event was detected, the start and end time of the projections used to build the corresponding µCT images were determined, resulting in a timespan for which corresponding AE signals registered by sensor AE_1_ (closest to the scanning position) were detected and divided into separate AE embolism datasets (Fig. 8). Also, the start and end time of the projections where no embolism formation was detected in consecutive events, breaks and runs were determined, resulting in a timespan corresponding to the non-embolism AE datasets (Fig. 8). At the end of the experiment, 457 embolized vessels were detected using the image processing procedure on the µCT data, for which the AE_1_ signals were divided into 132 embolism and non-embolism datasets (Fig. 8).

The open source software package Fiji for multidimensional scientific imaging was used to process the reconstructed 2D µCT cross-sections [55]. To automate the processing procedure, we used two custom-written Fiji macros. The first macro was developed to reduce noise, allowing a better comparison between images of consecutive events and runs, by cropping each image as close as possible to the contours of the cross section, and median filtering the cropped images in the *z*-direction (3D kernel of [51–53]) (Fig. 8). The second macro was used to compare µCT images, by registering images of consecutive time steps using bUnwarpJ to match their contours [56] and taking the difference between two registered consecutive images. The larger size of vessels over other xylem elements results in high absolute differences in corresponding pixels during the transition from water-filled vessels (grey pixel area on µCT image) to embolized vessels (black pixel area on µCT image) between two consecutive µCT images. The function ‘Find Maxima’, manually controlled by a threshold (set at 30), was used to differentiate between true embolism events and noise. Finally, the (x, y) coordinates of the detected embolized vessels were stored.

### Unsupervised and supervised machine learning

**Fig. 9:**
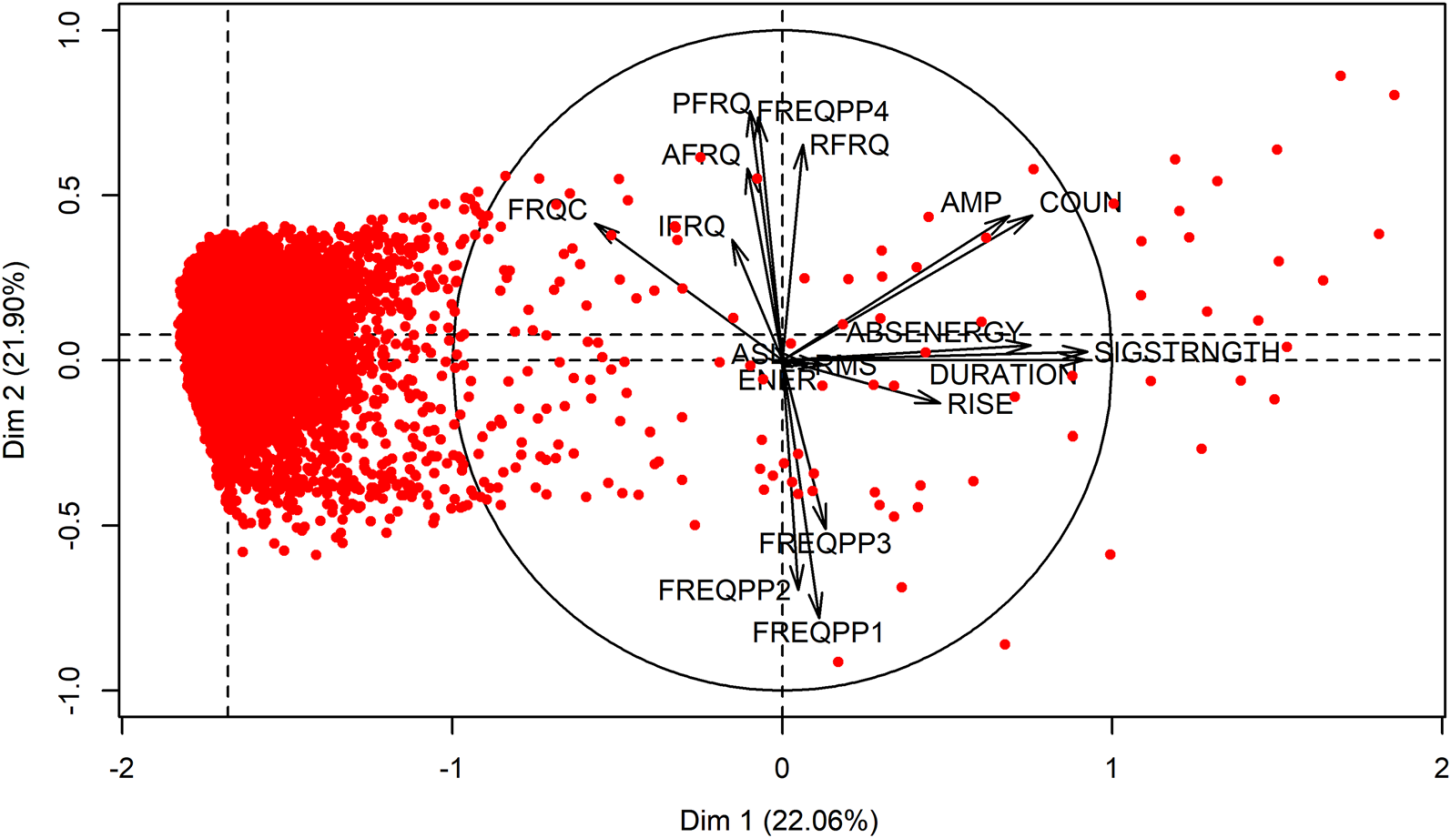
Individual AE signals (red) and AE waveform parameters (black arrows) factor map of the principal component analysis (PCA) executed on the entire AE dataset registered by sensor AE_1_ on *Fraxinus excelsior* L. during dehydration. Outliers separated from the major cluster correspond best with the number of embolism detected by µCT (457). AE waveform parameters ABSENERGY, DURATION, SIGSTRNGTH, COUN, and AMP (Table 4) are correlated with the first dimension explaining 22% of the data distribution, and AE parameter FREQPP2 (Table 4) negatively correlated with the second dimension explaining 22% of the data distribution

**Fig. 10:**
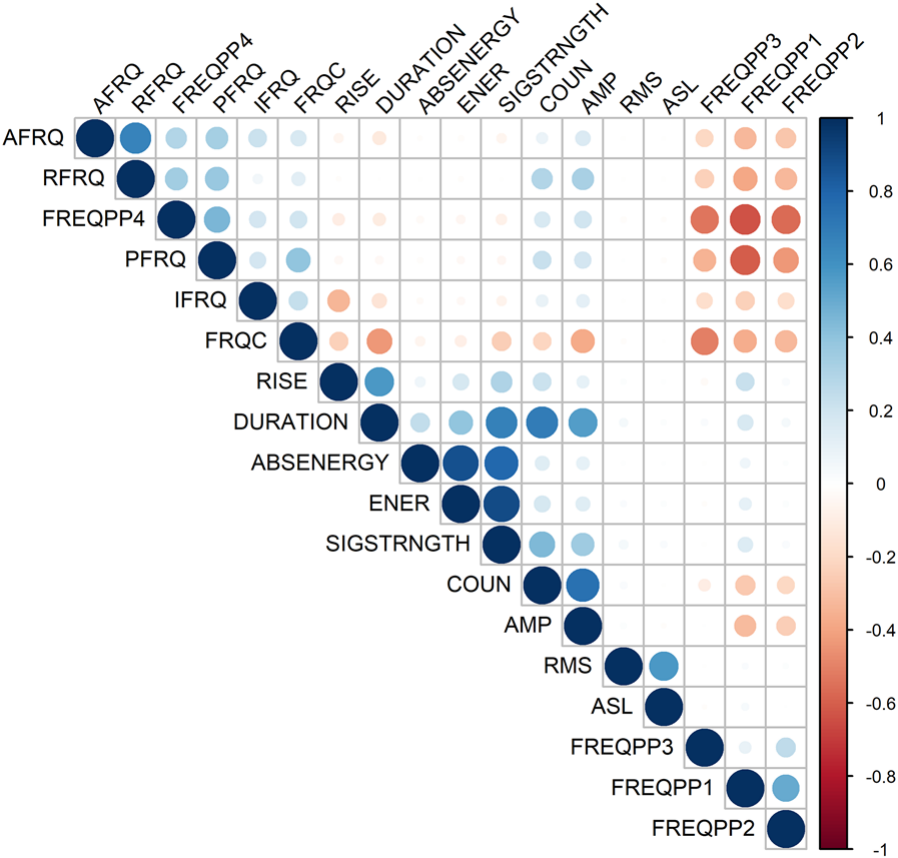
Correlation matrix correlogram of the 18 AE waveform parameters describing AE signals originating from the entire AE dataset registered by sensor AE_1_ on *Fraxinus excelsior* L. during dehydration. AE waveform parameters ABSENERGY, DURATION, SIGSTRNGTH, COUN, and AMP (Table 4) are positively correlated amongst themselves (blue gradient), and AE parameter FREQPP2 (Table 4) negatively correlated with COUN and AMP (red gradient)

To determine which AE sources are coupled to embolism events, the underlying distribution in recorded AE signals of sensor AE_1_ (closest to the µCT scanning point) was determined via principal component analysis (PCA) based on the 18 parameters describing each AE signal (Table 4), and was visualized by combining individual and variable factor map plots with the R package FactoMineR (Fig. 9) [57]. With a total of 25,901 registered AE signals, PCA illustrated that the vast majority of these points were present within a large cluster, while a lower number of signals were separated from the cluster as apparent outliers (Fig. 9). Because µCT detected a total of 457 embolism events, PCA indicated that these outliers were most likely the AE source related to embolism formation. In addition, the correlogram of the correlation matrix between the 18 AE waveform parameters was constructed to visualize the underlying correlations in order to decide which AE waveform parameters were sufficiently related to another to be used as variables in the further detection of embolism-related AE (Fig. 10).

Principal component analysis indicated that outliers in AE signals that were distinctively separated from the major cluster (Fig. 9) mainly followed the direction of the AE waveform parameters ABSENERGY, DURATION, SIGSTRNGTH, COUN, and AMP (Table 4). These parameters were positively and best (length of the arrows) correlated with the first principal component explaining 22.06% of the data distribution. The correlogram also illustrated that the AE waveform parameters ABSENERGY, DURATION, SIGSTRNGTH, COUN, and AMP were positively correlated among themselves (blue gradient) (Fig. 10). The AE parameter FREQPP2 was negatively and well correlated with the second principal component explaining 21.90% of the data distribution (Fig. 9), and negatively correlated to mainly AE waveform parameters AMP and COUN (red gradient) (Fig. 10). Because Vergeynst et al. [12] indicated FREQPP2 as important in clustering embolism-related AE, and to establish a link between parameters describing the shape of the AE signal and its frequency spectrum, FREQPP2 in addition to ABSENERGY, DURATION, SIGSTRNGTH, COUN, and AMP were selected for the consecutive machine learning steps to detect embolism-related AE from the total measured signals.

Based on the PCA results, with the six AE waveform parameters as possible sources for embolism formation (outliers Fig. 9), histograms of these parameters were constructed for five randomly selected (from a total of 132) embolism and non-embolism AE datasets, of which two per embolism and non-embolism datasets are shown (Fig. 2), to examine the efficiency of static thresholding to distinguish embolism formation from other AE sources.

Because determining histogram thresholds per AE parameter for the 132 separate AE datasets is too cumbersome, receiver operating curves (ROC) were constructed to determine which AE parameter yielded the most promising threshold on the entire AE dataset. With the interest in distinguishing embolism-related AE from other sources, a two-class prediction problem can be considered in this case, in which the outcomes are labeled either as positive (embolism) or negative (non-embolism). This means that there are four possible outcomes, but for the construction of the ROC curve only the true positive (TP, the actual embolism is predicted correctly, y-axis) and false positive (FP, a non-embolism is predicted as embolism, x-axis) rate are required. For each AE parameter, the TP versus FP rate enables to determine different static AE parameter thresholds. The first threshold in the ROC curve is the maximum value of each AE parameter over the entire dataset, typically resulting in solely a TP rate, but too strict to detect all the registered embolism events by µCT. Therefore, each maximum threshold is gradually adjusted and the number of AE signals in the embolism and non-embolism datasets is compared to the total number of embolism events detected by µCT. The most suited AE parameter to demarcate thresholds on the AE dataset to detect embolism-related AE is determined as the one for which the ROC curve stays as close as possible to the y-axis for the most stringent cut-points (i.e., as far as possible from the first bisector) and then deflects horizontally when the total number of embolism events registered by µCT (457) is reached.

Linear discriminant analysis (LDA) was used as supervised machine learning method to tackle the AE classification problem. The most straightforward strategy to tackle such a problem is to model the probability of an instance having a certain label given the feature vector *x*: $P(Y=y\mid X=x)$, which is called the posterior probability. Here *Y* is the random variable for the label and *X* the random variable modelling the features. Labeling an instance is done by assigning it the highest posterior probability, and if the posterior is modeled directly this is known as the discriminative approach (e.g., logistic regression). Using Bayes' rule, the posterior probability can be rewritten as:

1 $$P\left(Y=y\mid X=x\right)=\frac{P\left(X=x\mid Y=y\right)P(Y=y)}{P\left(X=x\right)}, $$

with $P(X=x\mid Y=y)$the likelihood of observing a feature vector *x* in an instance with a label *y*, *P*(*Y* = *y*) the prior of sampling an instance with a label *y*, and *P*(*X* = *x*) the evidence or the probability of encountering an instance with this particular feature vector. Note that it is not necessary to compute this last factor explicitly, as it is independent of the label. The label with the highest (arg max ()) posterior probability (y*) is predicted for a given feature vector *x* (Eq. 2):

2 $${y}^{*}={argmax}_{y}P\left(Y=y\mid X=x\right) $$

In generative models that generate both input and output variables, it is the likelihood and the prior that are modeled using the training data, in contrast to the posterior in the discriminative approach. The posterior probability is then only computed afterwards, using Bayes' rule. As the term generative implies, one can generate feature vectors associated with a given label. In practice, the model of the likelihood often does a poor job of modeling the conditional feature distribution, but can nevertheless give rise to good predictions. LDA is an example of a simple generative model, where every class is modeled by a normal distribution with the same covariance structure. In case of a binary classification problem, the features of the first class are distributed as $N({\mu }_{0},\Sigma )$ and of the second class as $N({\mu }_{1},\Sigma )$, with ${\mu }_{0}$ and ${\mu }_{1}$ the respective expected value of the feature vector within a class and $\Sigma$ the covariance matrix. The log-posterior of LDA also gives rise to a linear model.

In this study, ${\mu }_{0}$ represents the expected value of a feature vector of a measurement that is not an embolism event and ${\mu }_{1}$ the expected feature vector of a feature vector associated with an embolism event. Both are assumed to have the same covariance structure $\Sigma$. We have estimated ${\mu }_{0}$ and ${\mu }_{1}$ by taking a weighted average over the averages feature vector of each dataset. For ${\mu }_{0}$ and ${\mu }_{1}$, the weight for each data set is the number of non-embolism and embolism events, respectively, that were detected in a dataset. The global covariance matrix was computed in a similar way, after which a probability was attributed to each registered AE signal based on the weights calculated by the LDA model.

Linear discriminant analysis probabilities were summed for each AE dataset and the resulting sum was indicative for the expected number of AE signals classified as embolism-related (e.g., LDA on AE dataset 8 resulted in 44 embolism-related AE, Table 2). These were then used to construct the acoustic vulnerability curve. To determine whether the probability outcomes of the LDA model were suited to threshold the entire AE dataset, a ROC curve was constructed with the cut points representing a gradual decrease in maximum LDA probability (Fig. 3).

### Wood anatomy

**Fig. 11:**
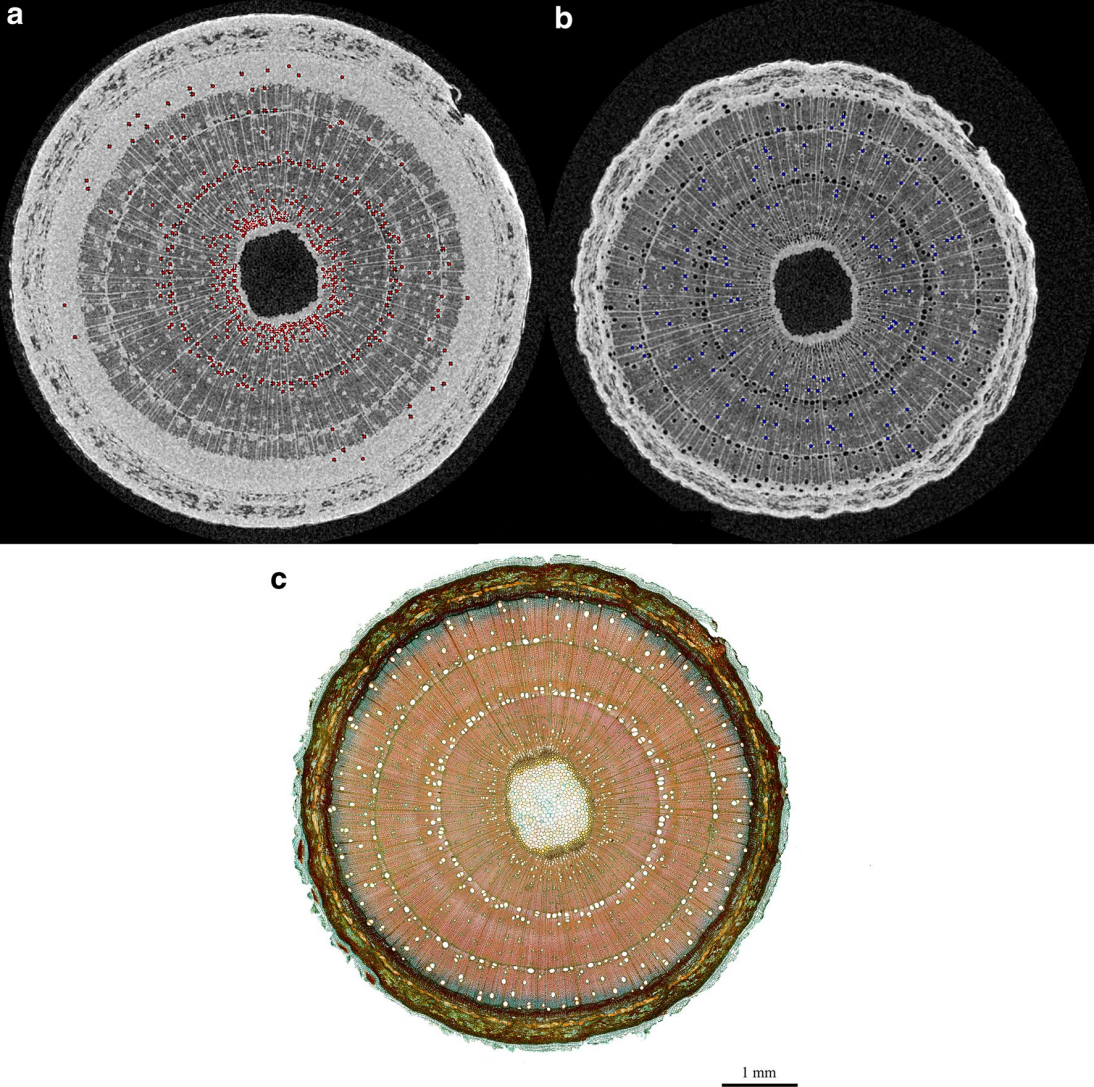
µCT image of *Fraxinus excelsior* L. at the start of the dehydration experiment (**a**), and at the end of the dehydration experiment (**b**). Native embolized vessels (**a**), and non-embolized vessels (**b**) are respectively highlighted in red and blue (**b**). Resolution = 7.5 µm. **c** Stained 35-µm thick cross-section of a 2-year-old *Fraxinus excelsior* L. stem at the µCT scanning point showing from inward to outward pit, xylem, cambium and bark. The cross section had a total of 1100 vessels. Scale bar = 1 mm. Credit Dr. Olivier Leroux

A wood sample of ~ 5 cm in length was taken from the scanned section, and included the marked position of scanning to perfectly match µCT images with the anatomical cross-section. The sample was preserved in a mixture of 70% ethanol (99%), 15% deionized water and 15% glycerol. A 35 µm thick cross section was cut from the sample at the exact point of scanning with a sliding microtome (Hn-40, Reichert-Jung, Saarland, Germany) at the Department of Biology, Ghent University. The cross section was stained for 15 min with 0.5% w/v astra blue, 0.5% w/v chrysoidine, and 0.5% w/v acridine red and mounted in euparal after dehydration in isopropyl alcohol. Images were captured using a Nikon Ni-U epifluorescence microscope equipped with a Nikon DS-Fi1c camera (Fig. 11c).

Anatomical analysis was restricted to manually counting the number of xylem vessels on the cross-section with the image analysis software Fiji. A total of 1100 vessels was obtained from the cross-section and was used to translate the number of embolized vessels derived from the µCT images to percentage embolism formation.

### Acoustic and µCT vulnerability curve

A total of 25,901 AE signals was registered by sensor AE_1_ and 90,416 by sensor AE_2_, which were per sensor cumulated over the measurement period and averaged over 10 min. The endpoint of the acoustic vulnerability curve (VC_AE_) is normally determined via the local maximum of the third derivative of cumulative AE [12], which was however not yet reached (Fig. 6), because not all vessels were embolized at the end of the dehydration experiment (Fig. 11b). Correct determination of non-embolized vessels on the µCT image was facilitated by the anatomical cross-section (Fig. 11c), which perfectly matched with the µCT scanning point. *Fraxinus excelsior* L. had a total of 1100 vessels, of which 541 were natively embolized (Fig. 11a), and 102 were not embolized at the end of the experiment (Fig. 11b).

Because complete embolism formation was not reached at the end of the dehydration experiment (Figs. 6, 11b), all registered AE signals had to be used in constructing the VC_AE_. The number of native embolized vessels was taken into account when converting absolute cumulative AE to percentage cumulative AE (%) following the assumption that VCs start from a fully hydrated condition [16]. The unfiltered cumulative AE of sensor AE_1_ and AE_2_ were translated to percentage cumulative AE by rescaling between 0 and 100% following the technique of Vergeynst et al. [12] (Fig. 6). For the dataset derived from sensor AE_1_, an additional VC_AE_ was constructed based on the LDA model output (VC_AE-E_). The LDA model detected 518 embolism-related AE signals, which were cumulated over the measurement period, averaged over 10 min, and for LDA rescaled from 0 to 96% as not all vessels were embolized at the end of the experiment (i.e., (541 + 518/1100)*100). The number of embolism formation events derived from the µCT scans was used to construct a µCT vulnerability curve (VC_CT_), which was also averaged over 10 min and rescaled between 0 and 91% (i.e., (541 + 457/1100)*100) to obtain percentage embolism formation (%).

The time axis of the different VCs was replaced with a continuous xylem water potential axis using the stress–strain curve. In this curve, point measurements of xylem water potential or stress (ψ_x_, MPa) are plotted against xylem shrinkage or strain (Δd/di, µm mm^−1^) measured with the dendrometer (see Additional file 3). A segmented-linear regression between ψ_x_ point measurements and continuous Δd/di with two breakpoints was obtained with the segmented R package [58] (see Additional file 3). The three linear regression equations were used to calculate the continuous xylem water potential values.

### Statistical analysis

A smooth spline function in the stats library in R software (RStudio version 1.1.419-© 2009–2017 RStudio, Inc.) was fitted to the vulnerability curves. Drought vulnerability values such as the onset of embolism formation (ψ_x_ at which 12% of embolism-related AE and µCT occur; AE_12_/CT_12_), 50% embolized (ψ_x_ at which 50% of embolism-related AE and µCT occur; AE_50_/CT_50_), full embolism (ψ_x_ at which 88% of embolism-related AE and µCT occur; AE_88_/CT_88_) and endpoint of the VCAE (ψ_x_ at which 100% of embolism-related AE occur; AE_100_) were determined [59]. Differences in VCs were quantified using the absolute difference in percentage embolism formation compared to the reference µCT VC.

## Supplementary information

**Additional file 1.** Photographic overview of the experimental setup of the* Fraxinus excelsior* L. tree in the custom-built holder with carbon fiber tube designed to ensure stable and centered positioning of the tree during µCT scanning (A). The tree is equipped with two broadband point-contact AE sensors in a pvc spring-containing holder to continuously register AEs from the progressively dehydrating xylem (AE_1_, AE_2_), and a point dendrometer to continuously register xylem shrinkage. This potentiometer-type of dendrometer was selected, as its working principle does not generate a magnetic field, which would otherwise produce acoustic interference.

**Additional file 2.** Waveform of a registered AE signal from a dehydrating * Fraxinus excelsior* L. stem. AE waveform parameters peak amplitude, rise time, counts from peak amplitude (several threshold crossings indicated with green boxes), wave energy (red) and duration from peak amplitude describe the shape of the AE signal.

**Additional file 3.** Stress-strain curve (black, open circles) between point measurements of xylem water potential (MPa) and xylem shrinkage (Δd/di, µm mm^−1^) of* Fraxinus excelsior* L. during dehydration. The segmented-linear regression with two breakpoints (black and red dashed line) divided the dataset in three linear regressions with their own equation and R2 (black, red, and grey) from which continuous xylem water potential was calculated.

## Data Availability

The datasets used and/or analyzed during the current study are available from the corresponding author on reasonable request.
