# Supplementary figures and images for: X-ray microtomography and linear discriminant analysis enable detection of embolism-related acoustic emissions
Source: Plant Methods. 2019 Dec 17;15:153. doi: 10.1186/s13007-019-0543-4 (PMC6916244; doi:10.1186/s13007-019-0543-4)

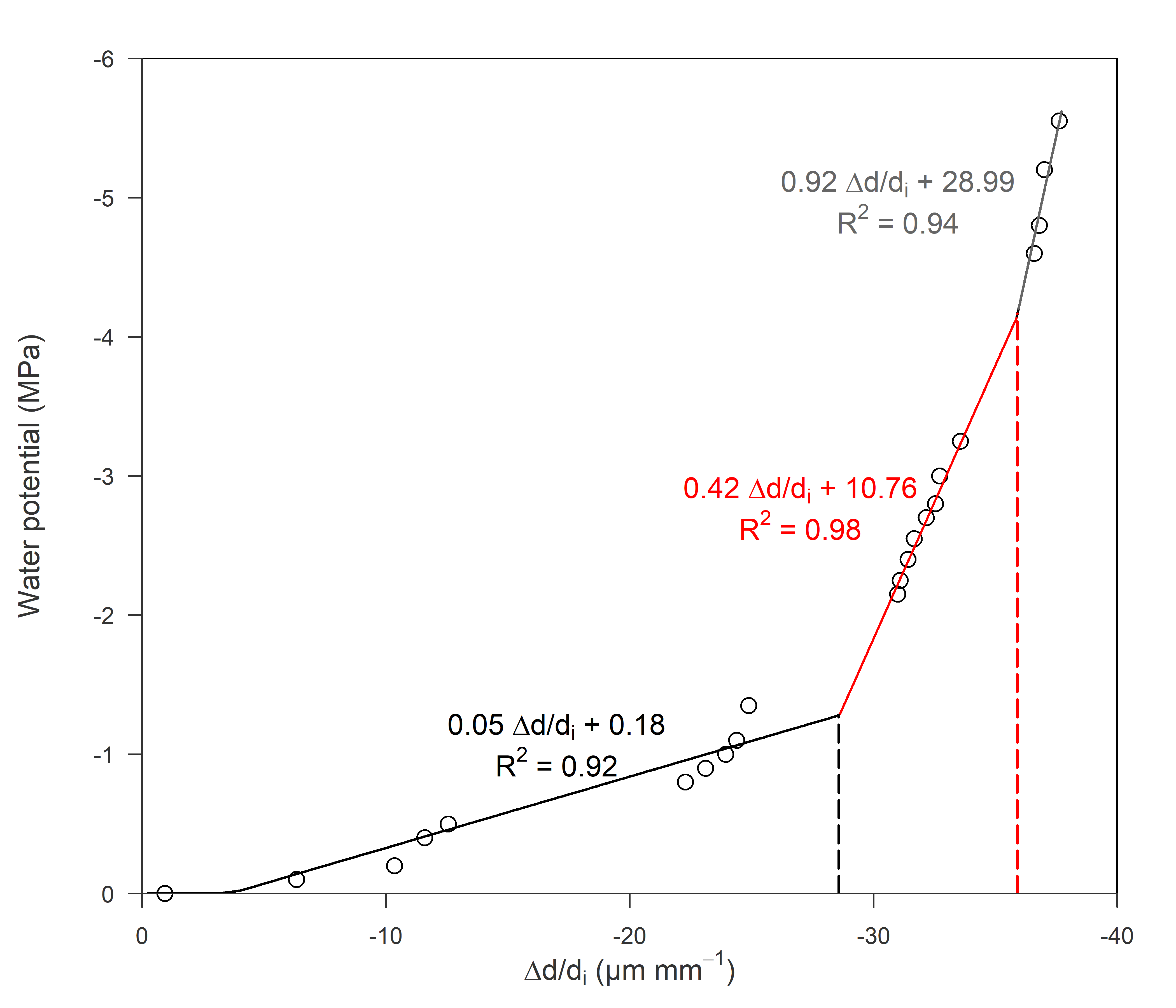

Supplement: Supplementary file 3 — Additional file 3. Stress-strain curve (black, open circles) between point measurements of xylem water potential (MPa) and xylem shrinkage (Δd/di, µm mm−1) of Fraxinus excelsior L. during dehydration. The segmented-linear regression with two breakpoints (black and red dashed line) divided the dataset in three linear regressions with their own equation and R2 (black, red, and grey) from which continuous xylem water potential was calculated. [file 13007_2019_543_MOESM3_ESM.tiff]
